# Eco friendly nanofluidic platforms using biodegradable nanoporous materials

**DOI:** 10.1038/s41598-021-83306-w

**Published:** 2021-02-15

**Authors:** Sungmin Park, Seongjun Hong, Junsuk Kim, Seok Young Son, Hyomin Lee, Sung Jae Kim

**Affiliations:** 1 Department of Electrical and Computer Engineering, Seoul National University, Seoul, 08826 Republic of Korea; 2 Department of Chemical and Biological Engineering, Jeju National University, Jeju, 63243 Republic of Korea; 3 Nano System Institute, Seoul National University, Seoul, 08826 Republic of Korea; 4 Inter-University Semiconductor Research Center, Seoul National University, Seoul, 08826 Republic of Korea

**Keywords:** Nanofluidics, Microfluidics

## Abstract

Splendid advancement of micro/nanofluidic researches in the field of bio- and chemical-analysis enables various ubiquitous applications such as bio-medical diagnostics and environmental monitoring, etc. In such devices, nanostructures are the essential elements so that the nanofabrication methods have been major issues since the last couple of decades. However, most of nanofabrication methods are sophisticated and expensive due to the requirement of high-class cleanroom facilities, while low-cost and biocompatible materials have been already introduced in the microfluidic platforms. Thus, an off-the-shelf and biodegradable material for those nanostructures can complete the concept of an eco-friendly micro/nanofluidic platform. In this work, biodegradable materials originated from well-known organisms such as human nail plate and denatured hen egg (albumen and yolk) were rigorously investigated as a perm-selective nanoporous membrane. A simple micro/nanofluidic device integrated with such materials was fabricated to demonstrate nanofluidic phenomena. These distinctive evidences (the visualization of ion concentration polarization phenomenon, ohmic/limiting/over-limiting current behavior and surface charge-governed conductance) can fulfill the requirements of functional nanostructures for the nanofluidic applications. Therefore, while these materials were less robust than nano-lithographically fabricated structures, bio-oriented perm-selective materials would be utilized as a one of key elements of the biodegradable and eco friendly micro/nanofluidic applications.

## Introduction

Since last two decades, nanofluidics has been drawn significant attentions due to its new physics and various applications that have never been demonstrated by microfluidics^1–3^. Such advances were originated from the unique surface effect of surface charge^4,5^, electrical double layer overlap^6,7^ or surface slippage^8,9^, etc. Those phenomena^10–12^ were extensively applied for novel engineering applications in the field of bio-/chemical-analysis^13,14^, environmental science^15^ and lab on a chip application^16^, etc*.*, showing superior performance to microfluidic applications’. To achieve these nanofluidic properties, nanofabrication techniques are essential step to be developed. In the early era of nanofabrication, sophisticated and expensive methods in high-class cleanroom should be utilized. Focused ion beam (FIB) and Nano imprint lithography (NIL) are the representative examples of such nanofabrication methods. In FIB (or E-beam) lithography, a focused beam of ions (or electrons) is employed to create a fine nanostructure by expensive beam-writing equipment in high-class cleanroom. NIL is relatively simple and cheap fabrication method using nanostructured mold. More recently, simpler nanofabrication methods using silicon bilayer^17^, nanoglassblowing^18^, diffractive photomask^19^, ion-track-etch^20^, thin film deposition^21^, hot embossing^21^ and stress release processing^21^ have been introduced, but, however, most of these methods still need the high-class clean room facilities and the substrates are not biodegradable.

Recently, soft lithographical methods with the minimal usage of cleanroom have accelerated the advances of nanofluidic studies and applications due to their cost- and time-efficiency^22,23^. Especially, elastomeric material-based lithography is one of the easiest way to fabricate the nanostructure. For example, nanochannels can simply be created by the roof-top collapse of elastomeric poly-dimethylsiloxane (PDMS) microchannel^24^. Similarly, when micro-patterns were covered by PDMS block, triangular nanochannels were created at both sides of the micro-pattern^25,26^. For scale-up or enhancing the nanofluidic effects, nanoporous medium which has intrinsic nanostructure (e.g. graphene oxide^27,28^, ionic hydrogel^29–31^, polymeric resin^23,32,33^, self-assembled nanoparticles^34,35^ and Nafion coated sponge^36^, etc*.*) could be utilized as well. Porous medium-based nanofabrications are not only cheap but also scalable so that they have been applied in various applications that may need high-throughput requirement^36^. Dielectric breakdown of the PDMS substrate was also used to create a nanojunction^37,38^. When the high electric field was applied between wedge-shaped PDMS microchannels, trapezoidal nanojunction was created.

Such endeavors finally reached to the low cost and disposable application, particularly useful for point-of-care devices in remote settings. Biocompatible materials have been actively employed for the building block in such device. For example, most of microfluidic functions were rebuilt with paper-based microfluidic platform recently^39–41^. Hydraulic (or mechanical) pumping in a conventional microfluidic system was appropriately replaced by capillary force of cellulose fibers^42^. For the visualization of the biological/chemical reaction, a colorimetric assay was implanted on a paper-based microfluidic device. Biomolecule preconcentrator^43^ and separator^44^ or energy harvesting system^45^ with a perm-selective membrane were also demonstrated in a paper-based micro/nanofluidic device. Especially, several studies have already suggested a paper-based nanoelectrokinetic platform which is operated by external electric field. Gong et al.^46^ conducted DNA analysis in the paper-based device, enabled by ion concentration polarization (ICP) for preconcentrating Hepatitis B virus DNA target. Phan et al.^40^ fabricated paper-based ICP device only using printed hydrophobic material such as wax. Han et al.^43^ developed paper-based preconcentrator with lateral-flow, achieving high preconcentration performance up to 1000-fold. Yang et al.^47^ fabricated paper-based ICP device incorporating straight and convergent channels. However, the choice of materials for the nanostructure is still limited within non-biocompatible materials, while few biocompatible materials were demonstrated with sophisticated treatments such as epoxy filling in wood-based nanostructure^48^ and the effective reduction of phytochemicals^49,50^. In the light of such needs, in this work, new biocompatible and biodegradable nanoporous materials originated from living organisms were utilized as a perm-selective material. Firstly, human nail or hen egg (albumen and yolk) were proven to be a proper candidate for the nanofluidic functionality. The direct measurement of counter-ionic species at the other side of the material could verify the perm-selectivity, but, however, one needs to consider the size of tracers. For example, small tracers such as basic blue 3^51^, creatinine^52^ and proton^53–56^ can pass through the materials, while large tracers such as Rhodamine 6G^57^, Alexa 488^57–59^, Sulforhodamine B^58,59^ and FITC^60–63^ can not. Thus, demonstrating the perm-selectivity of the material using a counter-ionic dye is less representative method due to the size dependency. Therefore, we experimentally demonstrated the perm-selectivity by showing (1) the direct visualization of ICP (i.e. depletion zone and enrichment zone), (2) Ohmic-limiting-over-limiting current behavior and (3) surface-charge-governed conductance. Regardless of experimental apparatus and the physicochemical parameter of chemicals used, the perm-selectivity must guarantee the appearance of (1), (2) and (3).

Lastly, nanoporous structures of egg were integrated with a paper-based micro/nanofluidic device to realize ICP phenomenon. Even though this fabrication method and used materials were less robust than nano-lithographical methods and substrates, bio-oriented perm-selective materials in this work could be applied for the fully bio-degradable micro/nanofluidic platforms which have significant potentials in terms of low-cost, eco friendly and fabrication easiness.

## Fabrication of nanofluidic device with bio-oriented materials

### Nail device

The first material for the bio-oriented nanostructure was the human nail because several literatures referred that the human nail is composed of keratinous tissue which enables the interesting mass transport^64^. The fact that a nail is gradually hydrated indicated that human nail is a porous medium^65^ and a number of studies have shown that various ions and biological molecules could pass through the nail plate by diffusion or electro-diffusion. In addition, the images of nail plate by Field-Emission Scanning Electron Microscope (Carl Zeiss, Germany) were shown in supporting information. The number of pores was visually counted from the SEM image of bottom view as ~ 40 nanopores in 20 µm^2^ area. In the meantime, we can experimentally measure the total resistance (*R*) of immersed nail plate as 2.35 × 10^6^ Ω. See supporting information for detailed conductance measurement method. The resistance equation, *R* = *ρ d*/*A* (*R* is the total resistance of immersed nail plate (2.35 × 10^6^ Ω) , *ρ* is resistivity of KCl 100 mM (0.766 Ωm), *d* is the thickness of nail plate (0.5 mm) and *A* is the total summation of the cross-sectional area of nanopores in nail plate) would give *A* = 163 µm^2^. Thus, there were 48 × 10^6^ nanopores (∵ (40 nanopores/20 µm^2^) × 24 × 10^6^ µm^2^), since the contacting area of the nail plate to the electrodes was 24 mm^2^. Thus, each nanopore had a radius (*r*_*n*_) of 1.04 nm (∵ 48 × 10^6^ × π *r*_*n*_^2^ = 163 µm^2^). While a literature reported the size as 0.7 nm^66^, the estimated size in this work was enough for the nail to possess a perm-selective property. Furthermore there were several literatures to support that ion transport in human nail highly depended on its physicochemical properties such as porosity, pore size, and surface charge^64^. The evidence suggest that pore structures in the human nail are nanoscale and can have nanoelectrokinetic properties that affect to ion transport through the human nail.

**Figure 1:**
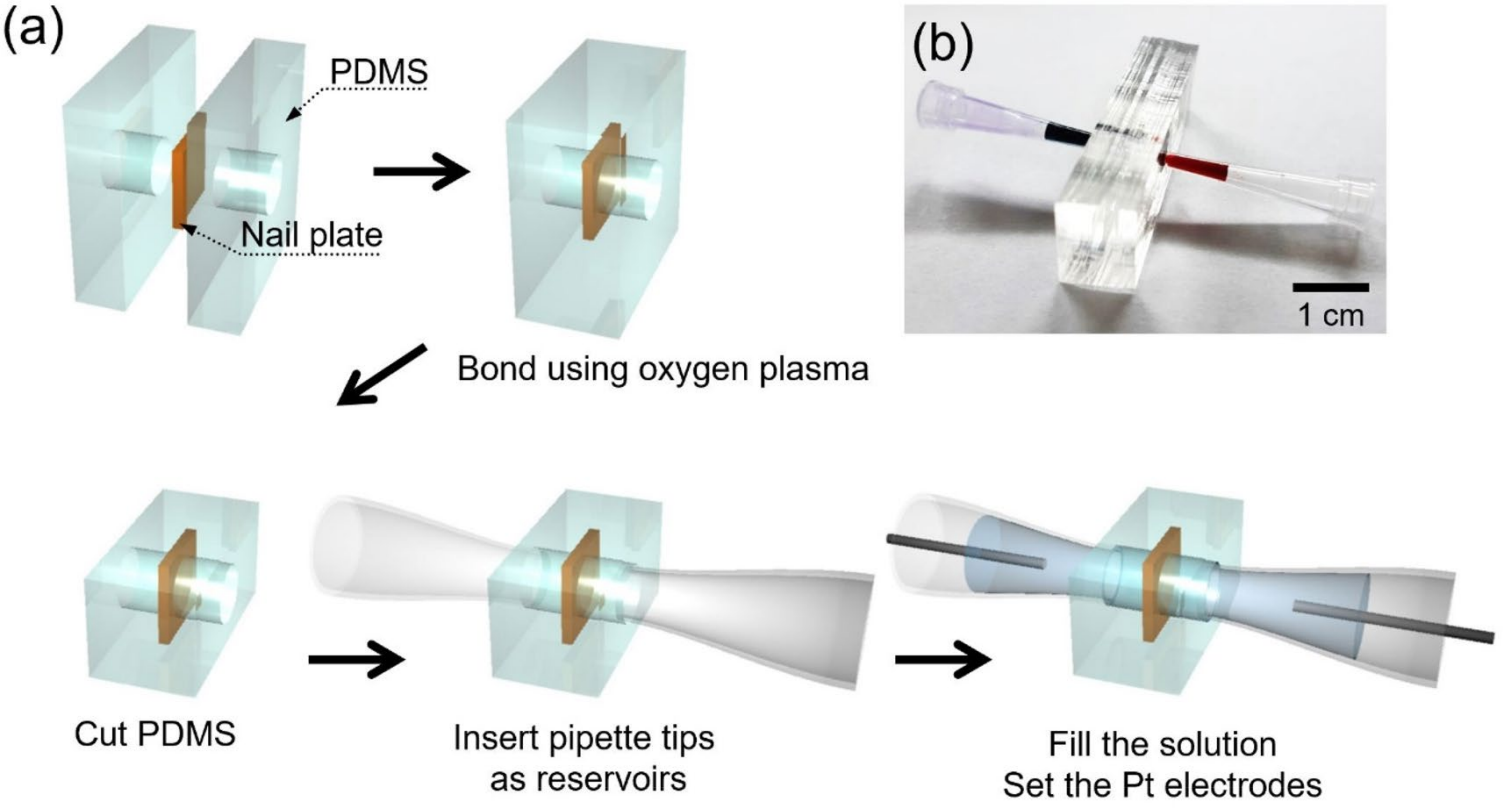
(**a**) The fabrication process of the nail device. (**b**) The photo of assembled nail device.

The fabrication process of the nail device was as follows. The main building block of the device was PDMS (Sylgard 184 silicone elastomer kit, Dow Corning, USA). PDMS base was mixed with a PDMS curing agent at 10:1 ratio and degassed in a vacuum chamber for 1 h. The mixture was poured on the petri dish until the depth of mixture was 0.5 cm and was cured in the oven for 4 h at 75 °C. The cured PDMS was detached from the petri dish and cut into two blocks which had the dimension of 5 cm (width) × 5 cm (height) × 0.5 cm (depth). The center of each block was punched by biopsy punch (BF-15F, Kai Medical, Japan) to create a hole (diameter: 1.5 mm) which was used as the channel of the device. The nail plate was prepared with dimension of 2 mm (width) × 2 mm (height). For cleaning the nail plate, it was washed by iso-propyl alcohol (sigma Aldrich, USA) in three times and wiped out by the dust free wipes (Kimtech science wipes, Kimberly-Clark, USA). After that, the nail plate was dried in room temperature at least 1 h. As shown in Fig. 1a, the nail plate was sandwiched between two PDMS blocks and then they were irreversibly bonded with oxygen plasma (CUTE-MP, Femto Science, Korea) treatment. Top side and the bottom side of the bonded device were cut to secure the focal length of an optical microscope for clear observation of the channel. The pipette tips as reservoirs were inserted to the hole of PDMS blocks. Assembled device was shown in Fig. 1b. Before the I-V measurements and visualization experiments of nail plate, 0.001X PBS solution at 25 °C was injected into the device and maintained at least 2 h for hydrating the nail plate. Then, new solution was refilled and we conducted the I-V measurements and visualization experiments. For conductance measurement of a nail plate, the nail plate was immersed in the KCl solution at each concentration ranging from 0.01 to 100 mM for 24 h as stated in supporting information. The experimental protocols (nail sampling and test) have been approved by the Seoul National University Ethical Review Committee (under the approval code, PBT-19-039) with the relevant guidelines and regulations of the standards set by the Declaration of Helsinki. A written informed consent document, for study participation, testing of nail samples and the publication of a potentially identifiable image in this article, was obtained from all subjects for samples. After the test, the nail plates were disposed according to the regulation of Institute of Environmental Protection and Safety at Seoul National University.

### Egg device

The second material was a hen egg. An albumen which is white part of an egg is similar to the physical morphology of a jelly like hydrogel. In addition, albumen has been used as a support medium in the electrophoresis of protein detection^67^ and as nanoporous mats because of its nanopores which can visually confirm by SEM image of previous studies^68,69^. The size of gel network of albumen was estimated as 100–150 nm^69^. Using void estimation method^70^, the diameter of nanopore in albumen is 60–90 nm. A yolk which is yellow part of an egg forms a particle-like powder when it is heated^71^. The particle size of yolk was reported to be ~ 200 nm^72^. Using the void calculation again, the nanopores in denatured yolk was calculated as 120 nm, which is appropriate for the requirement of perm-selective material.

**Figure 2:**
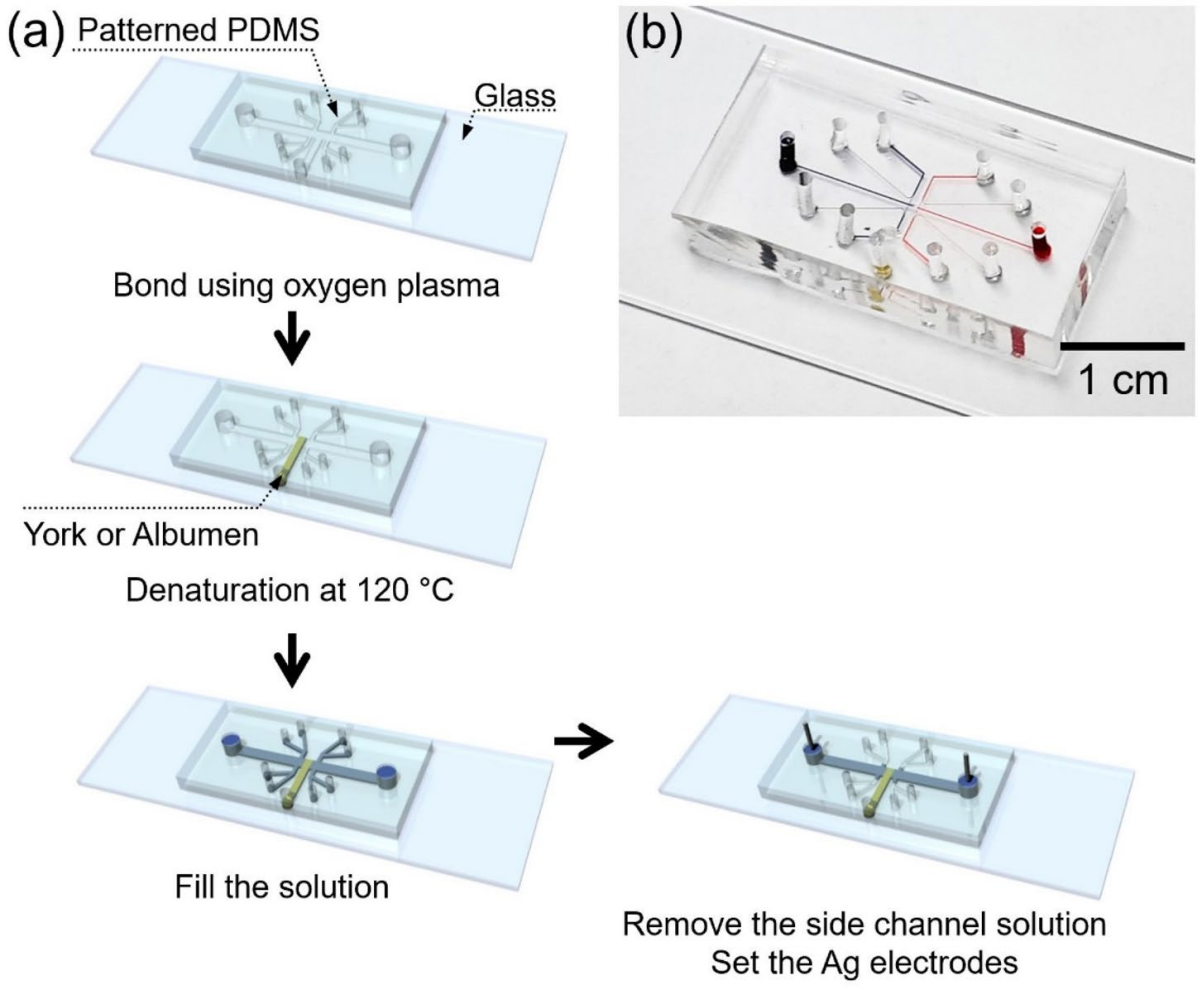
(**a**) The fabrication process of the egg device. (**b**) The photo of assembled egg device.

The fabrication process of egg device was as follows. Albumen and yolk were used as a nanoporous material. A T-shaped microchannel (100 um (width) × 15 um (depth)) together with air-valve microchannels for experimental easiness^73^ was fabricated by a general PDMS fabrication method as shown in Fig. 2a. After punching access holes (diameter: 1.5 mm) in the PDMS block, the T-shaped microchannel and glass slide were irreversibly bonded with oxygen plasma treatment. Then, albumen or yolk was injected into the microchannel up to the predetermined boundary and the device was heated at 120 °C for complete denaturation. This heating process could be considered as a sterilization process as well. Denatured egg completely blocked in the middle of the T-shaped microchannel so that one can have microchannel-nanojunction(egg)-microchannel configuration. After heating, pipette tips served as reservoirs were inserted into the access hole of the PDMS block. Two Ag electrodes were inserted into the reservoirs for applying an external voltage. The device was immersed in KCl solution (1 mM) for preconditioning. The fabricated egg device was shown in Fig. 2b.

## Materials and experimental setups

For visualization experiments with the nail device, PBS solution (0.001X) with Alexa 488 fluorescent dye (1 uM) was used as an electrolyte. Note that Alexa 488 is pH-insensitive dye^74^ so that a titration is not required. After injecting the solution into the device, one should wait at least 2 h so that the nail plate was sufficiently hydrated with the solution^75^. 100 V of DC bias was applied to the device by a source measure unit (SMU, Keithley 238, USA) via Pt electrodes at the reservoirs. SMU was calibrated by manufacture’s policy. The propagation of ICP layer was captured by an inverted fluorescent microscope (IX53, Olympus) and CellSens program.

For the I-V measurement with the nail device, PBS solution (0.001X) without fluorescent dye was used. The voltage was swept from 0 to 100 V at 1 V/min. The current values at each step were recorded by customized Labview program. I-V characteristics were measured five times in a device and more than 5 devices were tested. For conductance measurement of the human nail plate, it was measured three times for one concentration value so that totally 15 measurements were conducted for a device.

For visualization experiments with the egg device, KCl solution (1 mM) with Alexa 488 fluorescent dye (1 µM) was used as an electrolyte. 30 V of DC bias was applied to the device. The propagation of ICP layer was captured by the same experimental setup.

For the I-V measurement with the egg device, KCl solution (1 mM) without fluorescent dye was used. The voltage was stepwisely applied from 0 to 20 V at 0.1 V/sec. The current values at each step were recorded by customized Labview program. I-V characteristics were measured five times in a device and more than 20 devices (including both albumen and yolk) were tested. For conductance measurement of the denatured egg, it was measured five times for one concentration value so that totally 15 measurements were done for a device. The measurements were repeated with 4 devices for each albumen and yolk.

## Results and discussions

### The formation of an ion depletion zone in a nail device

When DC bias is applied through a perm-selective nanoporous media which is immersed in an electrolyte solution, this electric field induces the asymmetric ionic flux through the perm-selective media, causing an ion concentration imbalance^76^. The typical behavior of ions with the cation-selective membrane is that ions are depleted at an anodic side (i.e. ion depletion zone) and are enriched at a cathodic side (i.e. enrichment zone) of the media^61,77^. This phenomenon is called ICP or concentration polarization (CP) phenomenon^78,79^. In other words, the presence of the ICP phenomenon (or appearance of an ion depletion zone at an anodic side) near the media is the key evidence that the media has nanoscale structures.

**Figure 3:**
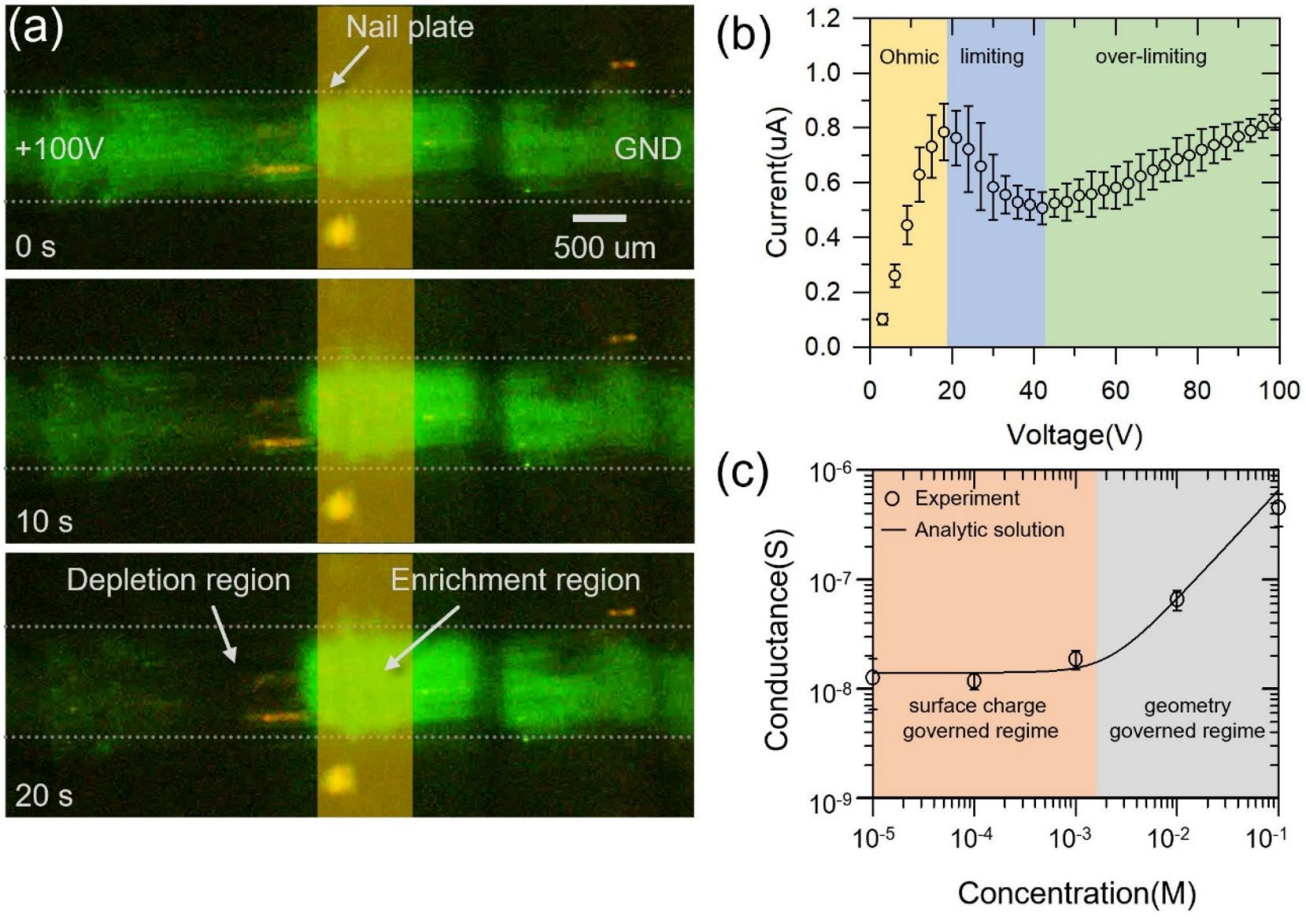
(**a**) The visualization of an ion depletion zone and an ion enrichment zone resulted from ICP phenomenon using nail device. (**b**) I-V characteristics of nail device. (**c**) Conductance profile of the nail device. The error bars on the plots were standard deviation.

As shown in Fig. 3a, negatively charged fluorescent dye (Alexa 488) was depleted at the anodic side with 100 V DC bias in the nail device. The concentration of fluorescent dye (1 uM) inside the solution was sufficiently low compared to that of major carriers (PBS 0.001X) so that the fluorescent dye rarely disturbed the device and it just used for tracer of the major carrier^30,80^. Therefore, the depletion region of the fluorescent dye represented the existence of ion concentration gradient near the anodic side of the nail plate, which confirmed that the human nail is not only a medium of mass transfer but also has the perm-selectivity. Also, the enrichment region was developed at the cathodic side as well. These facts strongly supported that the nail plate can be utilized as a cation-selective material. Note that the fluorescent signal can be exaggerated in Fig. 3a due to the shallow focal depth so that the nail seemed to be transparent, but actual nail plate was not transparent.

### I–V characteristics of the nail device

I–V characteristics in Fig. 3b showed typical linear sweep voltammetry of nanofluidic device which associated with a perm-selectivity. Without a perm-selective material, I-V characteristic of the device should show only a linear ohmic response. However, I-V responses through a perm-selective material are divided in three distinctive regions; Ohmic, limiting and over-limiting^6,61,81,82^. In the Ohmic region between 0 and 20 V, current linearly increased as a function of voltage as shown in Fig. 3b because the device had constant conductance until ICP was initiated. The following region is called the limiting region. In theory, the development of an ion depletion region near an anodic side of a perm-selective material leads the decrease of conductance of the device so that current values would be saturated. In realistic conditions (i.e. the sweep rate was not slow enough), a peak current was appeared before saturation^83,84^ called an overshoot effect. As shown in Fig. 3b, a peak current was observed around 20 V, and after that, current decreased shortly (i.e. limiting current region). In ideal cases, the current can be constantly maintained. As the voltage increased further, however, the over-limiting region appeared. In over-limiting region, current value increased as the voltage increased shown in the Fig. 3b because of various electrokinetic mechanisms such as surface conduction^85–88^, electroosmotic convection^61,89^ and electroosmotic instability^81,90^. These I-V characteristics are the fingerprint that the nail plate is a perm-selective material.

### Conductance profile of the nail plate

The conductance curve in the nano-structures as a function of electrolyte concentration differs from the behavior of the typical meso-structures. In the case of the meso-structure, the conductance through the structure is linearly proportional to the concentration of the bulk electrolyte solution because the concentration inside the structure is equal to the concentration of electrolyte solution outside the structure. However, in the case of the perm-selective nano-structure, the conductance through the structure becomes invariant under a threshold electrolyte concentration, called “surface charge governed regime.”^4,25,91^ This constant conductance region reflects that the surface charge of the structure dominantly affects the ion concentration inside the structure at a low electrolyte concentration. In the meantime, the conductance turns to a linear profile over the threshold concentration, called “geometry governed regime.” As shown in Fig. 3c, the nail plate should be a perm-selective material, since the conductance profile of it followed the conductance profile of conventional perm-selective material. See supporting information for the fabrication and conductance measurement method of the nail device.

The value of surface charge of the human nail can be obtained from this conductance profile because the surface charge relates to the conductance of the material. The conductance (*G*_*mat*_) was theoretically calculated as^91,92^

1 $$ G_{mat} = \alpha \left[ {\mu_{ + } \left( {\sqrt {4c_{0}^{2} + N_{w}^{2} } + N_{w} } \right) + \mu_{ - } \left( {\sqrt {4c_{0}^{2} + N_{w}^{2} } - N_{w} } \right)} \right] $$

where *α* is empirical coefficient, µ_+_ and µ_−_ are the electrophoretic mobility of cation and anion, respectively, and *c*_0_ is the bulk electrolyte concentration. *N*_*w*_ is the surface charge concentration (i.e. Donnan concentration) which is defined as

2 $$ N_{w} = - \frac{{2q_{s} }}{{Fr_{n} }} $$

where *q*_*s*_ is the surface charge density, *F* is the Faraday constant and *r*_*n*_ is the equivalent hydrodynamic radius of the nanopores inside the material^93^. Here, experimentally obtained *r*_*n*_ was 1.04 nm, which was close to the literature value of 0.7 nm^66^. See supporting information for detailed estimation. *α* and *N*_*w*_ were obtained from fitting curve of the conductance profile (Fig. 3c). In this case, *α* and *N*_*w*_ were estimated as 2.1 × 10^–2^ C⋅m/mol and 4.35 ± 1.45 mM, respectively. Using these values*,* the surface charge of the nail plate was calculated as—0.22 ± 0.07 mC/m^2^ and it was well-agreed with the literature value (~ 0.1 mC/m^2^)^66^. While this value was lower than the surface charge of silicon (− 15 mC/m^2^) and Nafion (~ − 200 mC/m^2^)^32^, the nail plate has a perm-selectivity, and therefore, it was proven to be used for perm-selective material again.

### The characteristics of the egg device

**Figure 4:**
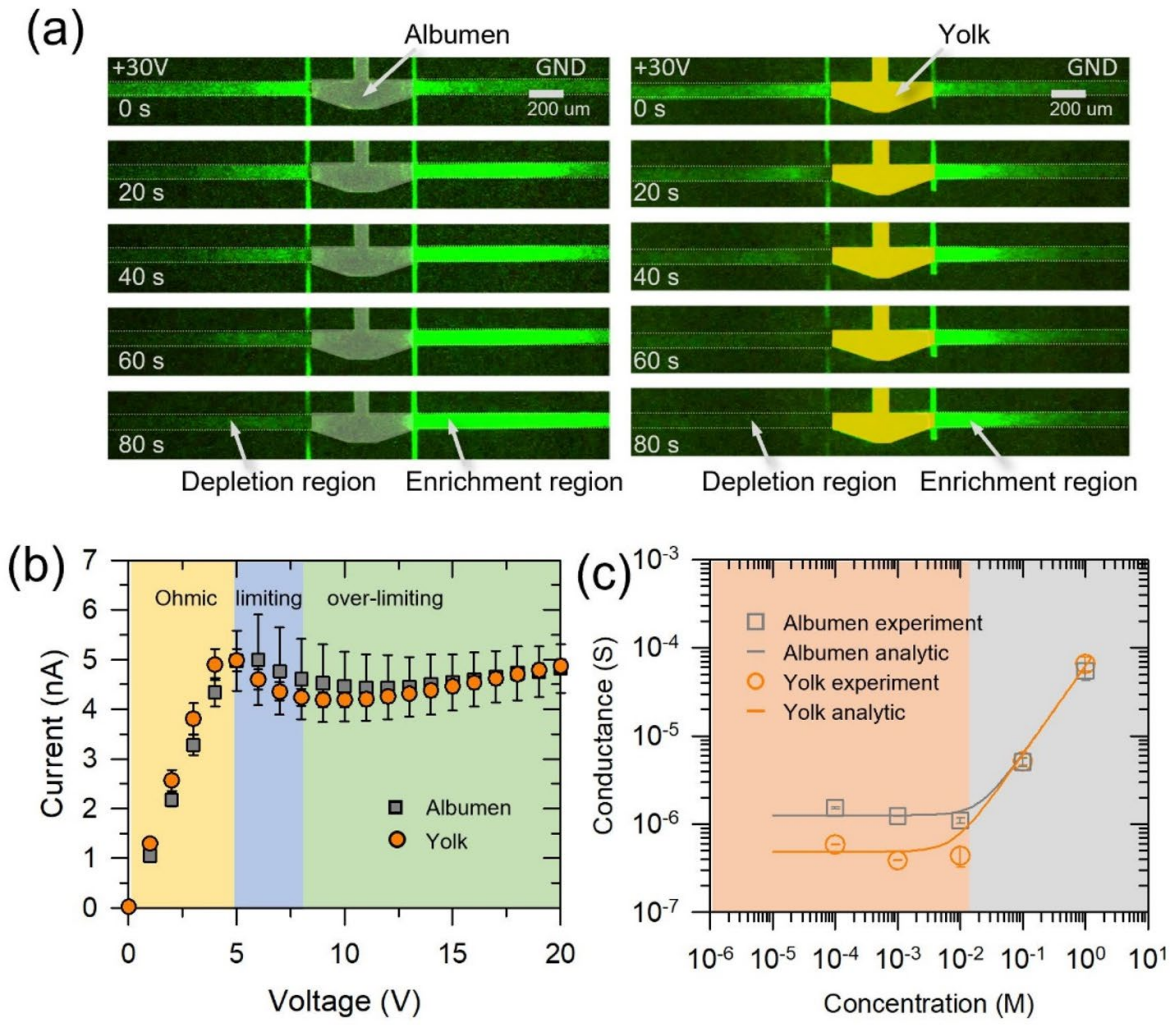
(**a**) The visualization of an ion depletion zone and an ion enrichment zone resulted from ICP phenomenon using egg devices. (**b**) I-V characteristics of egg devices. (**c**) Conductance profile of the egg devices. The error bars on the plots were standard deviation.

The same procedures were repeated with the egg device in this section. The external DC bias of 30 V was applied to the device. As shown in Fig. 4a, the ion depletion zone and the ion enrichment zone were formed at the anodic and cathodic side of egg material, respectively, which led to the conclusion that albumen and yolk are not only a medium of mass transfer but also a perm-selective nanoporous material.

Also, the devices had the unique I-V responses as similar as the nail device as shown in Fig. 4b. In this device, external voltage was swept from 0 to 20 V at 0.1 V/sec. Interestingly, similar I-V characteristics were observed in both cases of albumen and yolk, since egg device had long microchannel and short nanojunction, meaning that the ohmic loss inside the microchannel was dominant in such low concentration of 1 mM^94^. Thus, the similar Ohmic responses of both albumen and yolk were observed in Fig. 4b. Importantly, the egg albumen and yolk experienced a limiting current and over-limiting current as well, leading to another strong evidence.

The quantitative properties of albumen and yolk material were analyzed by similar manner in previous section. As shown in Fig. 4c, the conductance-concentration plot of albumen and yolk material also showed two distinguishable regimes. See supporting information for the fabrication and conductance measurement method of the egg device. The conductance value of albumen in surface charge governed regime was higher than that of yolk as shown in Fig. 4c. This means that *N*_*w*_ of albumen was higher than that of yolk. *α* was estimated by 0.2 C⋅m/mol in both albumen and yolk. *N*_*w*_ of albumen and yolk were estimated as 41.5 ± 8.5 mM and 16.0 ± 3.0 mM, respectively. (Equivalent hydrodynamic radius, *r*_*n*_, of albumen and yolk was assumed as 30 nm and 60 nm, respectively^68,70,72^.) Using the value of *N*_*w*_*,* the surface charge of albumen and yolk were estimated as—60.1 ± 12.3 mC/m^2^ and—46.3 ± 8.7 mC/m^2^, respectively. These values were higher than the surface charge of silicon (− 15 mC/m^2^) so that the perm-selectivity of albumen and yolk is comparable with silicon nanochannels.

### ICP demonstration using egg nanojunction in paper device

ICP phenomenon in a paper-based micro/nanofluidic device using hen egg albumen and yolk was demonstrated in this section. Paper consisted of micron-size celluloses can usually be used as a microchannel because it has not only micro-sized holes, but also it has a capillary force to spontaneously absorb a sample fluid^39,42^. Since denatured hen egg albumen and yolk have nanopores, micro/nanofluidic phenomenon can be occurred in this paper-based device, if albumen and yolk were integrated into the paper.

**Figure 5:**
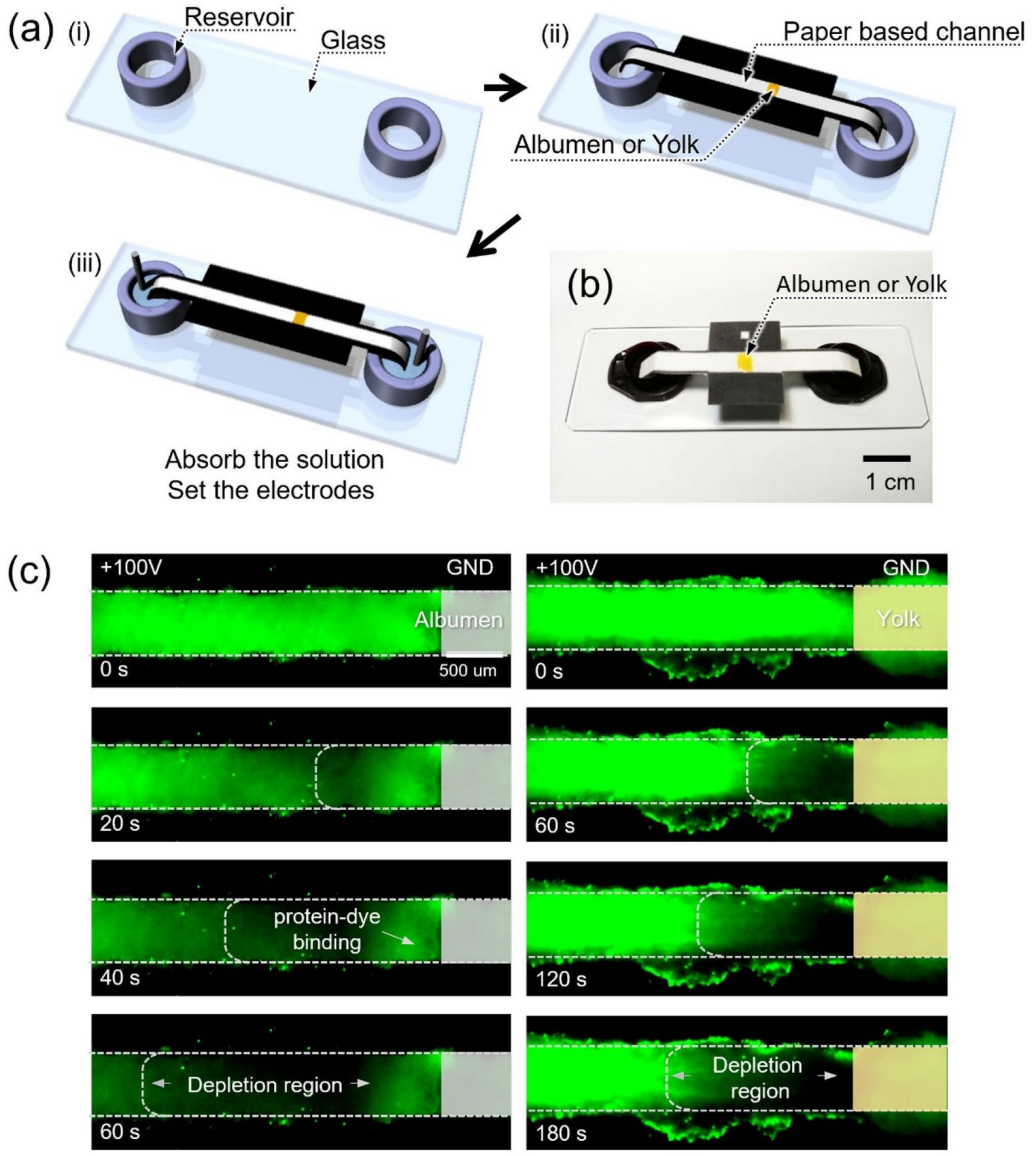
(**a**) The fabrication process of the paper based ICP device using egg membrane. (**b**) The photo of assembled paper based ICP device. (**c**) The visualization of ICP phenomenon using paper based ICP device.

The paper-based ICP device was fabricated using cellulose paper (180 µm of thickness and a mean pore diameter of 3 µm, Whatman grade 1, Sigma-Aldrich, USA) incorporated with egg nanojunction as follows. Figure 5a-i: 10 mL-tube caps were glued on a slide glass as a supporter. Figure 5a-ii: A commercial wax printer (ColorQube 8570, FUJI Xerox) was utilized for printing hydrophobic guide of microchannel (the black part of paper). Then, albumen or yolk was dropped at the center of paper for forming a cation-selective nanojunction. After resting 5 min for complete absorption into the paper, the patterned paper was heated at 120 ºC for 1 min. The fabricated devices were then dried in petri dishes with a cover at room temperature. Figure 5a-iii: Ag electrodes were inserted into both reservoirs and connected to a power supply (Keithley 236, Keithley Instruments, USA). Fabricated device was shown in Fig. 5b. For the visualization experiments with the paper device, KCl (1 mM) with Alexa 488 fluorescent dye (1 uM) were used as an electrolyte and a tracer, respectively. After dropping the electrolyte solution on the paper, one needed to wait until the paper was completely wetted by capillary force. 100 V of DC bias was applied to the device. The propagation of ICP layer was captured by an inverted fluorescent microscope and CellSens program.

As shown in Fig. 5c, fluorescent dye molecules were depleted from both albumen and yolk, confirming that ICP phenomenon occurred in these paper-based devices. However, in the case of albumen, fluorescent dye molecules in the immediate vicinity of the nanojunction were not depleted as shown. This unexpected fluorescent signal could be explained by protein–dye binding. Anionic florescent dyes (Alexa 488) used in this work, strongly bind to heat-denatured ovalbumin which is the main protein found in egg albumen, making up approximately 55% of the total protein^95^. Since the cross-sectional area of the paper device was much larger than that of PDMS device, binding effect only occurred in paper device and had been neglected in PDMS device. Nevertheless, the depletion region was observed near both albumen and yolk nanojunction, so that these paper-based ICP devices could be utilized for the element of the biodegradable and eco-friendly applications.

## Conclusions

Most of the conventional nanostructures in a micro/nanofluidic applications have been made of non-biocompatible materials and fabricated by sophisticated facilities in high-class cleanroom facilities. On the other hand, biocompatible materials such as paper or woven thread in microfluidic applications were recently reported. Because of this gap, novel nanofabrication using a functional and biodegradable materials should be required to design a low-cost and eco friendly micro/nanofluidic applications. In this work, human nail plate and denatured hen egg albumen and yolk as a functional biomaterial were introduced as a perm-selective nanoporous material. These materials were originated from the living organisms unlike most of the previously reported nanoporous materials.

The perm-selectivity of these materials was verified by ICP phenomenon, limiting/over-limiting current behavior and unique surface charge governed conductance profile. In the meantime, quantitative properties such as surface charge density and Donnan concentration of these materials were obtained as summarized in Table 1.

**Table 1:** Comparisons of surface charge density and Donnan concentration of nail plate, albumen and yolk with other materials.

	Nail	Albumen	Yolk	Nafion	Silicon nanochannel
|*q*_*s*_|(mC/m^2^)	0.22 ± 0.07	60.1 ± 12.3	46.3 ± 8.7	~ 200	~ 15
*N*_*w*_ (mM)	4.35 ± 1.45	41.5 ± 8.5	16.0 ± 3.0	~ 720	Depending on geometry

Their physicochemical properties were less robust than solid-state material, since the bio-oriented material has been grown in different environments such as species of chicken and health condition of human nail, etc. Therefore, the error bars of the plots in this work were relatively large, but we confirmed that they have a necessary perm-selectivity for nanoelectrokinetic applications. In order to minimize variations, the device should be carefully sealed for improving the repeatability. In the case of nail device, there could be the concerns about false positive or inconclusive results, if the nail plate was used in point-of-care applications. Instead of applying to such applications, however, our nail study can provide the important information about drug delivery through human nail plate. For the efficient treatment of the disease such as onychomycosis, etc., a number of researchers have tried to reveal the drug permeation rate through nail plate^96,97^, while electrokinetic properties and perm-selectivity of nail plate have not been deeply studied. Thus, in this work, we presented the quantitative electrokinetic properties of the nail plate such as surface charge density, Donnan concentration and its cation-selectivity so that these quantities can guide to researchers who try to develop the drug penetration for nail disease. In the case of the egg device, high applied voltage or repeated usage may destroy the nanostructure.

Although these materials have unavoidable limitations such as contamination from nail donor, low surface charge, anionic molecules binding with egg albumen, low repeatability and short shelf life, they have great potentials for fully biodegradable low-cost micro/nanofluidic devices in remote settings.

## Supporting information

Device fabrication and measurement for surface charge and pore size estimation

## Supplementary Information

Supplementary Information
