# Supplementary material for: Eco friendly nanofluidic platforms using biodegradable nanoporous materials
Source: Sci Rep. 2021 Feb 15;11:3804. doi: 10.1038/s41598-021-83306-w (PMC7884701; doi:10.1038/s41598-021-83306-w)
Supplement: Supplementary file 1 — Supplementary Information [file 41598_2021_83306_MOESM1_ESM.docx]

Eco friendly Nanofluidic Platforms using Biodegradable Nanoporous Materials

Sungmin Park^*^, Seongjun Hong^*^, Junsuk Kim, Seok Young Son,
Hyomin Lee and Sung Jae Kim^†^

**SI Note 1. Device fabrication for conductance measurement**

For conductance measurement of a nail plate, a nail was cut as the dimension of 12 mm (width) × 2 mm (height) × 0.5 mm (thickness). The nail plate was immersed in the 9 mL DI water for 24 hours to hydrate it. Then, it was immersed in the 9 mL KCl solution at a concentration ranging from 0.01 mM to 100 mM for 24 hours as shown in SI Figure 1. After taken out and wiped off, crocodile clips were connected to both faces of the nail plate and the voltage was applied from -10 V to 10 V at the sweep rate of 2 V/sec. The current values at each step were recorded by customized Labview program. The conductance values were extracted from the slope of the I-V curve (Ohmic regime) as shown in Figure 3(c). At 100 mM condition where an electrical double layer overlap could be negligible, the total resistance (*R*) of the immersed nail plate was obtained as 2.35 × 10^6^ Ω.

**SI Figure 1.** Schematic diagram of the conductance measurement experimental process of the nail plate.

The fabrication of the device using egg for conductance profiles was as follows. PDMS block which had a rectangular hole (15 mm × 10 mm) and the thickness of 1.5 mm was irreversibly bonded with glass slide using O_2_ plasma to fabricate container as shown in SI Figure 2. Aluminum foil was glued at the edge (10 mm side) of the container by epoxy to apply electric potential uniformly. Egg yolk or albumen was poured in the container and heated at 120 °C for complete denaturation. Then, the device was immersed in the KCl solution at a concentration ranging from 10^-4^ M to 1 M for more than 24 hours. At each concentration, the voltage was swept from -0.2 V to 0.2 V at 0.04 V/sec increments. The current values at each voltage value were recorded by customized Labview program. The conductance was then calculated from the slope of the I-V curve in Ohmic regime.

**SI Figure 2**. The schematic diagram of the conductance measurement experimental process of the egg devices and the photo of assembled egg conductance measurement devices

**SI Note 2. Estimation of nanopore size in nail plate by SEM**

The images of a nail plate by Field-Emission Scanning Electron Microscope (Carl Zeiss, Germany) were shown in SI Figure 3. The number of pores were visually counted from the SEM image of bottom view as ~40 nanopores in 20 μm^2^ area. In the meantime, we can experimentally measure the total resistance (*R*) of immersed nail plate as 2.35 × 10^6^ Ω in SI Note 1. The resistance equation, *R* = *ρ* *d* / *A* (*R* is the total resistance of immersed nail plate (2.35 × 10^6^* *Ω) , *ρ* is resistivity of KCl 100 mM (0.766 Ωm), *d* is the thickness of nail plate (0.5 mm) and *A* is the total summation of the cross-sectional area of nanopores in nail plate) would give *A* = 163 μm^2^. Thus, there were 48 × 10^6^ nanopores (⸪ (40 nanopores / 20 μm^2^) × 24 × 10^6^ μm^2^), since the contacting area of nail plate to the electrodes was 24 mm^2^. Thus, each nanopore had a radius (*r_n_*) of 1.04 nm (⸪ 48 × 10^6^ ×  *r_n_*^2^ = 163 μm^2^). While a literature reported the size as 0.7 nm^1^, the estimated size in this work was enough for the plate to possess a perm-selective property.

**SI Figure 3.** SEM images of a human nail plate.

In the case of the egg yolk, the particle of yolk was reported as 200 nm^2^. Using the void calculation method^3^, the nanopores in denatured yolk is calculated as 120 nm, which is enough size for the requirement of perm-selective material. Secondly, the gel network of egg albumen had been reported as 100 – 150 nm^4^. Using void estimation again, the size of nanopore in albumen is 60 - 90 nm. This value was also enough size for the requirement of perm-selective material.

**References**

1 Murthy, S. N. & Maibach, H. I. *Topical nail products and ungual drug delivery*. (CRC press, 2012).

2 Phillips, G. O. & Williams, P. A. *Handbook of food proteins*. (Elsevier, 2011).

3 Roozbahani, M. M., Borela, R. & Frost, J. D. Pore size distribution in granular material microstructure. *Materials* **10**, 1237 (2017).

4 Croguennec, T., Nau, F. & Brule, G. Influence of pH and salts on egg white gelation. *J. Food Sci.* **67**, 608-614 (2002).
